# Screening of *Klebsiella pneumoniae* Isolates for Carbapenemase and Hypervirulence-Associated Genes by Combining the Eazyplex^®^ Superbug CRE and hvKp Assays

**DOI:** 10.3390/antibiotics12060959

**Published:** 2023-05-25

**Authors:** Jürgen Rödel, Yvonne Pfeifer, Martin A. Fischer, Birgit Edel, Sylvia Stoll, Wolfgang Pfister, Bettina Löffler

**Affiliations:** 1 Institute of Medical Microbiology, Jena University Hospital, Friedrich Schiller University, 07747 Jena, Germany; birgit.edel@med.uni-jena.de (B.E.); sylvia.stoll@med.uni-jena.de (S.S.); bettina.loeffler@med.uni-jena.de (B.L.); 2 Division Nosocomial Pathogens and Antibiotic Resistance, Department of Infectious Diseases, Robert Koch Institute, 38855 Wernigerode, Germany; pfeifery@rki.de (Y.P.); fischerm@rki.de (M.A.F.); 3 Department of Hospital Hygiene, Sophien-und Hufeland-Klinikum, 99425 Weimar, Germany; w.pfister@klinikum-weimar.de

**Keywords:** *Klebsiella pneumoniae*, carbapenemases, hypervirulence, LAMP

## Abstract

The acquisition of hypervirulence-associated genes by carbapenemase-producing *Klebsiella pneumoniae* is being increasingly observed, and easy-to-use diagnostic tests are needed for the surveillance of the hypervirulent *K. pneumoniae* (hvKp). In this pilot study, 87 *K. pneumoniae* isolates from invasive infections collected in 2022 and 2023 were analysed using the LAMP-based eazyplex^®^ Superbug CRE and hvKp assays for the simultaneous identification of carbapenemases and virulence genes (*rmpA/A2*, *iuC*, *iroC*, *ybt*, *clb*). Nine isolates showed a Kleborate virulence score of 4 or 5 (10.3%). The time for the results of the eazyplex^®^ assays ranged from 6.5 to 13 min, and the total turnaround time, including sample preparation, was less than 30 min. Five isolates, three of which produced New Delhi metallo-beta lactamase (NDM), were subjected to whole-genome sequencing (WGS) analysis for further characterisation. The eazyplex^®^ test results for beta-lactamase and virulence genes were confirmed. The eazyplex^®^ hvKp, currently only available as a Research Use Only assay, may be a useful tool for the rapid identification of hvKp without significant additional workload when combined with the eazyplex^®^ Superbug CRE assay for the detection of carbapenemases.

## 1. Introduction

As a leading cause of hospital-associated infections, *Klebsiella pneumoniae* has attracted particular attention, not only because of the increase in beta-lactam resistance but also because of the combination of broad-spectrum antibiotic resistance with the presence of hypervirulence-associated genes in several strains [1]. The increasing emergence of hypervirulent *K. pneumoniae* (hvKp) strains producing carbapenemases in Europe has led to a risk assessment by the European Centre for Disease Prevention and Control (ECDC) that carbapenem-resistant hvKp can cause difficult-to-treat or untreatable invasive infections in both immunocompromised patients and previously healthy people and that surveillance is needed to prevent further spread in healthcare settings [2].

*K. pneumoniae* strains are most commonly classified as hypervirulent when they possess multiple siderophores, specific capsule types, a hypermucoviscous phenotype, and are isolated from severe invasive infections [3]. The hvKp strains can belong to different sequence types (ST), however, a large cluster is of ST23, which expresses the K1 capsule [2]. Recently, outbreaks by hvKp expressing carbapenemases have been observed in regions with previously low incidence [4,5,6,7,8]. Of particular concern is the identification of strains with hybrid plasmids that harbour both beta-lactamases and virulence genes [3,9]. It is likely that hvKp is currently underdiagnosed. The ECDC risk assessment also includes a call for the early detection of such strains by testing strategies to be established in routine diagnostics [2]. In our laboratory, *K. pneumoniae* isolates are routinely screened for the presence of carbapenemases using the eazyplex^®^ Superbug CRE assay (Amplex Diagnostics, Gars-Bahnhof, Germany), a rapid molecular test based on loop-mediated isothermal amplification (LAMP) [10]. The aim of this proof-of-principle study was to investigate the performance of the eazyplex^®^ hvKP Research-Use-Only (RUO) assay (Amplex Diagnostics) targeting the hypermucoviscosity-associated genes *rmpA/A2*, colibactin, and the siderophores aerobactin, yersiniabactin, and salmochelin, for a combined rapid testing of carbapenemase- and virulence-associated genes in invasive *K. pneumoniae* isolates.

## 2. Results

A total of 87 *K. pneumoniae* isolates collected in 2022 and 2023 were analysed for this pilot evaluation study. This collection consisted of 43 isolates from blood cultures and 42 isolates from other specimens, including joint punctates, cerebrospinal fluid, abscesses, deep tissue, gallbladder fluid, urine, sputum, and bronchoalveolar lavage. Two carbapenemase-producing isolates from rectal screening swabs were also investigated. The eazyplex^®^ superbug CRE assay identified 11 isolates producing carbapenemases OXA-48 (*n* = 4), NDM (*n* = 6), and NDM+OXA-181 (*n* = 1).

**Figure 1:**
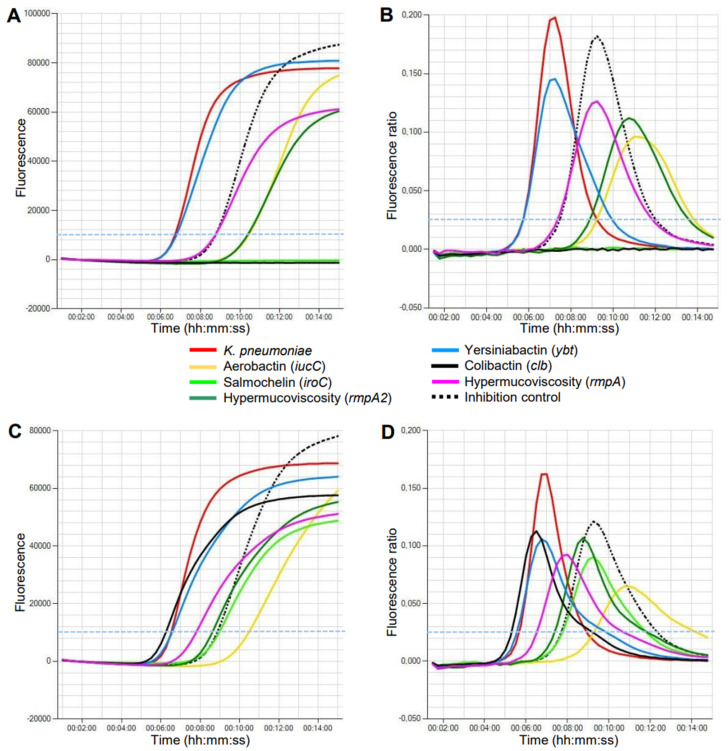
Detection of *K. pneumoniae* virulence genes by LAMP on the Genie HT instrument within 15 min. (**A**,**B**) Isolate 1 of ST395 with a virulence score of 4. (**C**,**D**) Isolate 7 of ST23 with a virulence score of 5. The thresholds of fluorescence level (**A**,**C**) and amplification rate (**B**,**D**) are marked with a broken light blue line.

The Kleborate virulence score has been proposed to classify *K. pneumoniae* by the presence of genes encoding multiple siderophores and colibactin (see Materials and methods) [1]. The eazyplex^®^ hvKp assay showed that 14 out of 87 isolates (16.1%) had an elevated virulence score ≥2 (Table 1). Nine of these scored 4 or 5 (10.3%). It should be noted that this rate does not reflect the true prevalence in our hospital setting, as not all *K. pneumoniae* isolates during the study period were included in this study. Of the 11 carbapenemase-producing *K. pneumoniae*, six isolates (54.5%) with the carbapenemases NDM (*n* = 4) and OXA-48 (*n* = 2) had an increased virulence score. All six isolates were also positive for the extended-spectrum beta-lactamase *bla*_CTX-M1_ group. In comparison, none of the carbapenemase-producing isolates collected between 2012 and 2021 and tested here had a Kleborate score ≥2. Only yersiniabactin, which is also common in the classical *K. pneumoniae* strains, was present in 66.7% of the isolates. The time for the results of the eazyplex^®^ hvKp assay, defined by the threshold time of fluorescence intensity, ranged from 6.5 to 13 min for the specific targets (Table 1). Representative amplification curves are shown in Figure 1.

The hypermucoviscosity of the colonies was assessed by the ‘string test’, which was positive for 7 of 14 isolates. Hypermucoviscosity-associated genes *rmpA/A2* were detected in nine isolates (Table 1).

The genomes of five isolates selected as representative examples based on virulence scores of 4 and 5 were sequenced. Subsequent MLST analysis revealed that isolates 1 and 2 with virulence scores of 4 belonged to ST395 and ST395-1LV, respectively. The positive eazyplex^®^ results for the NDM and CTX-M-1 groups were verified as NDM-1 and CTX-M-15 using Kleborate, and the identification of virulence factors could be confirmed (*rmpA/A2*, *iuc1*, and *ybt16*). They possessed the KL2 capsule type (*wzi 2* genotype). The ST395 strains have been observed in an increasing number of NDM-producing *K. pneumoniae* in Germany since 2022, however, the cgMLST analysis revealed no close genetic relationship between these isolates and isolates 1 and 2 in the present study [7]. Both isolates were obtained from the blood cultures of septic patients with cirrhosis. MLST analysis of the NDM-positive isolate 3, cultured from a joint aspirate from a patient with a periprosthetic infection, identified ST2096. The isolate possessed NDM-1 and the KL64 capsule type (*wzi 2* genotype). The virulence score of 4, as calculated from the eazyplex^®^ results, was confirmed by the identification of *ybt14* and *iuc1*. The string test of this isolate was negative, although *rmpA/A2* was detected. All NDM-producing isolates had a low MIC of <1/4 mg/L for aztreonam–avibactam and were susceptible to cefiderocol (Table 1).

Isolates 7 and 8, which were susceptible to third-generation cephalosporins and carbapenems but identified as hvKp with a virulence score of 5, were also further characterised by WGS. MLST demonstrated that they belonged to ST23, the archetype of hvKp [2,11]. No beta-lactamase genes could be detected, except for an intrinsic *bla*_SHV-11_ gene, but the presence of virulence-associated genes was confirmed (*rmpA/A2*, *iuc1*, *ybt1*, *iro1*, and *clb2*). As detected by PCR, both isolates possessed the K1 capsule type (*wzi 1* genotype) and the mucoviscosity-associated gene A (*magA*), which contributes to K1 capsule polysaccharide synthesis. Isolate 7 was obtained from a blood culture from a patient with urosepsis. Isolate 8 was cultured from a cerebrospinal fluid sample from a patient who developed meningitis following otitis media. Interestingly, *rmpA/A2* genes were confirmed using classical PCR/Sanger sequencing and the eazyplex^®^ hvKp assay in all five genomically characterised isolates, however, the Kleborate tool failed to detect any *rmpA* genes in isolates 3 and 7.

## 3. Discussion

There is a need for rapid assays that can be integrated into daily routine diagnostics for the early detection and surveillance of hvKp [2,4]. Diagnostics should not be restricted to carbapenem-resistant isolates. Non-carbapenem-resistant hvKp can also cause severe invasive infections, and carbapenemase-producing *K. pneumoniae* can acquire hypervirulence-associated genes by plasmid transfer and the formation of hybrid plasmids combining both characteristics on a single transferable unit [1,3,12]. This suggests that the diagnostic focus needs to shift from the detection of antimicrobial resistance alone to a broader strategy that includes the identification of virulence factors in order to limit the spread of high-risk *K. pneumoniae* clones and to improve timely clinical decision-making within antibiotic stewardship programmes [13,14,15].

The results of this study demonstrate for the first time that a newly developed LAMP-based assay for the testing of several hvKp-associated virulence genes can be easily combined with the commercial eazyplex^®^ Superbug CRE assay, which has already been evaluated as a reliable tool for the rapid identification of beta-lactamases [10].

There is currently no consensus on the definition of hvKp. Hypervirulence can generally be defined as the overexpression of capsular polysaccharides combined with the acquisition of more than one siderophore [1]. It should be noted that not only capsular serotypes K1 and K2 are associated with hvKp [1]. The ‘string test’ was not sufficient for the reliable screening of hvKp when compared to the detection of the capsular regulators *rmpA/A2*, which corresponds to the findings of other studies [1]. However, it has to be considered that the detection of *rmpA/A2* does not prove capsule overexpression, as both genes can be truncated [1,3,6]. The Kleborate score used is only based on the detection of various siderophores and colibactin. Aerobactin is typically associated with hvKp in combination with yersiniabactin [1]. Additional suitable markers are colibactin, a genotoxin that causes interstrand DNA cross-linking and subsequent double-strand breaks, and salmochelin, which is not used in the Kleborate score [16].

Treatment options for carbapenemase-producing *K. pneumoniae* are very limited. Ceftazidime–avibactam is often used for OXA-48 and KPC-producing isolates and cefiderocol for infections with metallo-beta-lactamase (MBL)-producing strains [17]. The combination of aztreonam and avibactam may also be active against MBL strains, however, they are not currently available and are in phase III clinical trials [18]. Alternatively, ceftazidime–-avibactam is now often combined with aztreonam for the treatment of MBL-producing strains [19].

Whole-genome-sequencing (WGS)-based analyses are increasingly being used to identify and characterise hvKp strains on demand, and they allow phylogenetic investigations for surveillance purposes. The combined presence of several resistance and virulence determinants in several isolates in the present study is of concern, and future analyses will show whether these genes are located on hybrid plasmids, facilitating their spread. As WGS is time-consuming, expensive, and requires extensive computing, simple and rapid methods to detect hvKp in routine diagnostic workflows are of interest. The results of this proof-of-principle study show that the eazyplex^®^ hvKp, currently only available as an RUO assay, can be a useful tool for the rapid identification of hvKp when combined with the eazyplex^®^ Superbug CRE assay for the detection of carbapenemases. There is no significant additional work, as a single sample preparation can be used for both assays. For the surveillance of hvKp, testing of *K. pneumoniae* isolates from both invasive infections and carbapenemase-positive screening samples may be appropriate. LAMP is suitable for screening virulent strains, and WGS can be used for confirmation.

## 4. Materials and Methods

### 4.1. Bacterial Strains and Antimicrobial Susceptibility Testing

This study included *K. pneumoniae* isolates collected between January 2022 and April 2023. They were isolated from clinical samples sent to the Clinical Microbiology Laboratory of the University Hospital Jena as part of routine patient care at the Jena University Hospital and the Weimar Hospital. Out of 2140 isolates, 87 were selected for this pilot study on the basis of isolation from blood cultures, invasive infections, or the presence of carbapenemases. In addition, 25 *K. pneumoniae* strains collected between 2012 and 2021 and positive for OXA, KPC, NDM or VIM genes were retrospectively analysed for the presence of virulence genes. Isolates were stored by cryopreservation at −80 °C using the CRYOBANK^®^ system (Mast Diagnostica, Reinfeld, Germany). For recultivation, one bead was immediately removed from the vial without thawing the entire sample and transferred to a solid medium. The identity of isolates in long-term storage was always verified by species identification and testing for the presence of carbapenemase genes after thawing.

Bacteria were cultured on Columbia sheep blood agar and Drigalski lactose agar (Thermo Fisher Scientific, Wesel, Germany). Single colonies were identified as *K. pneumoniae* using MALDI-TOF mass spectrometry (Vitek MS, bioMeriéux, Nürtingen, Germany) and examined for a hypermucoviscous phenotype using the ‘string test’, which is positive if a viscous string of >5 mm is obtained by stretching a colony on the agar plate using an inoculation loop. Antimicrobial susceptibility testing was performed by determining minimal inhibitory concentrations (MICs) using Vitek 2 (card N430, bioMérieux). For carbapenemase-producing isolates, the MICs of meropenem, ceftazidime–avibactam, and aztreonam–avibactam were determined using the MICRONAUT-S Labor Berlin MDR MIC/GN microtiter system (Merlin Diagnostika, Bornheim, Germany; distributed by Sifin Diagnostics, Berlin, Germany). The MIC of cefiderocol was determined with a gradient test strip (Liofilchem, Roseto degli Abruzzi, Italy; distributed by Bestbion, Köln, Germany). The breakpoints were interpreted according to the criteria of the European Committee on Antimicrobial Susceptibility Testing [EUCAST, v13.0, https://www.eucast.org/clinical_breakpoints (accessed on 3 January 2023)].

### 4.2. Eazyplex^®^ LAMP Assays

The eazyplex^®^ hvKp RUO assay (Amplex Diagnostics) is a ready-to-use test strip containing lyophilized master mixes with primers for *K. pneumoniae*, *iucC*, *iroC*, *ybt*, *clb*, *rmpA*, *rmpA2*, and an inhibition control in each well. The eazyplex^®^ superbug CRE IVDR assay (Amplex Diagnostics) contains master mixes with primers for *bla*_CTX-M-1 group_, *bla*_CTX-M-9 group_, *bla*_KPC_, *bla*_OXA-48_, *bla*_NDM_, *bla*_VIM_, and an inhibition control. A single colony of the isolates was suspended in 500 μL of resuspension and lysis fluid (RALF buffer, Amplex Diagnostics) and boiled for 2 min. After centrifugation at 1500× *g* for 1 min, 25 μL of the supernatant was added to each tube of the eazyplex^®^ test strip. Tests were run on a Genie HT machine (Amplex Diagnostics) at 65 °C for 20 min. Amplification was measured by real-time fluorescence detection using an intercalating dye contained in the lyophilized master mix of the assays. Test results were automatically calculated and reported by the integrated eazyReport™ software (Amplex Diagnostics) on the Genie HT instrument (Amplex Diagnostics). The virulence scores were assessed according to the Kleborate tool (https://github.com/katholt/Kleborate, (accessed on 3 January 2023)) as follows: 0, none present; 1, *ybt* only; 2, *clb* without *iucC* (regardless of *ybt*); 3, *iucC* only; 4, *iucC* and *ybt* without *clb*; 5, *iucC*, *ybt*, and *clb*. Increased virulence was defined when a score of at least 2 was reached [1].

### 4.3. WGS Analysis and PCR

For WGS, the isolates were grown in a Brain Heart Infusion (BHI) broth (BD, Heidelberg, Germany). The DNA was extracted from overnight cultures using a DNeasy Blood and Tissue Kit (Qiagen, Venlo, The Netherlands), according to the manufacturer’s instructions. A Qubit dsDNA HS Assay Kit (Thermo Fisher Scientific) was used for DNA quantification. Sequencing libraries were prepared by applying a Nextera XT DNA Library Prep Kit (Illumina, San Diego, CA, USA). Sequencing was performed on an Illumina NextSeq 550 using a v2.5 chemistry kit (2 × 150 bp), as described in the manufacturer’s protocol. The generated fastq-files were quality checked and trimmed using the Trimmomatic software with the following parameters: ILLUMINACLIP:NexteraPE-PE.fa:2:30:10 LEADING:3 TRAILING:3 SLIDINGWINDOW:4:15 MINLEN:36 [20]. De novo assembly was performed using a Unicycler v0.4.8, including the SPAdes assembler v3.13.01 [21,22]. The generated contigs were further analysed using Kleborate (https://github.com/katholt/Kleborate, (accessed on 3 January 2023)) and AMRfinder plus (https://github.com/ncbi/amr, (accessed on 3 January 2023)) [23]. Core genome multilocus sequence typing (cgMLST) was performed using SeqSphere+ v8.4.1 with the respective published MLST and cgMLST schemes [24]. Raw read data were assigned to ENA Accession No. PRJEB61660 (https://www.ebi.ac.uk/ena/browser/home, (accessed on 3 January 2023)). All isolates that were selected for genome sequencing were also tested for the hypermucoviscosity-associated genes *magA* and *rmpA* using the primers published by Yeh et al., as well as *rmpA2* (primer FWD, 5′-TTATGTGCAATAAGGATGTT-3′; primer REV, 5′-CTAGGTATTTGATGTGCAC-3′) [25].

## Figures and Tables

**Table 1:** *K. pneumoniae* isolates with increased virulence scores, identified by the eazyplex hvKp assay.

No.	Source	ST	String Test	Eazyplex LAMP Results, Threshold Time (min)						Virulence Score	Carbapenemase, ESBL ^a^	Antimicrobial Susceptibility Profile MIC (mg/L), Interpretation ^b^				
				*rmpA*	*rmpA2*	*iucC*	*iroC*	*ybt*	*clb*			CTA ^c^	MER ^c^	CTV ^c^	AZA ^c^	CID ^c^
1	Blood culture	395-1LV	+	8.75	10.5	10.5	−	6.75	−	4	NDM, CTX-M1 group	>64, R	32, R	>32/4, R	≤1/4, S	1, S
2	Blood culture	395	+	8.5	10.75	11	−	7	−	4	NDM, CTX-M1 group	>64, R	32, R	>32/4, R	≤1/4, S	1, S
3	Joint aspirate	2096	−	8.5	10.75	11	−	7	−	4	NDM, CTX-M1 group	>64, R	64, R	>32/4, R	≤1/4, S	1, S
4	Rectal swab	N.D. ^d^	−	−	13	11.75	−	6.5	−	4	NDM, CTX-M1 group	>64, R	32, R	>32/4, R	≤1/4, S	0.19, S
5	Rectal swab	N.D.	−	−	−	12.5	−	7.5	−	4	OXA-48,CTX-M1 group	>64, R	32, R	1/4, S	≤1/4, S	0.38, S
6	Urine	N.D.	−	−	−	14	−	−	−	3	OXA-48,CTX-M1 group	>64, R	32, R	≤1/4, S	≤1/4, S	0.19, S
7	Blood culture	23	+	7.75	8.5	10.5	9	6.5	6.25	5	−	≤1, S	≤0.25, S	N.D.	N.D.	N.D.
8	Cerebrospinal fluid	23	+	8.5	10.5	10	9	6	6	5	−	≤1, S	≤0.25, S	N.D.	N.D.	N.D.
9	Gluteal abscess	N.D.	+	7.75	13	12	8.25	6.75	6	5	−	1, S	≤0.25, S	N.D.	N.D.	N.D.
10	Foot gangrene	N.D.	−	−	−	13.75	-	8.25	−	4	−	1, S	≤0.25, S	N.D.	N.D.	N.D.
11	Sputum	N.D.	+	7.5	8.75	8.5	7.75	−	−	3	−	≤1, S	≤0.25, S	N.D.	N.D.	N.D.
12	Blood culture	N.D.	+	8	−	11.25	9.25	−	−	3	−	≤1, S	≤0.25, S	N.D.	N.D.	N.D.
13	Blood culture	N.D.	−	−	−	7.75	−	−	−	3	−	≤1, S	≤0.25, S	N.D.	N.D.	N.D.
14	Intra-abdominal abscess	N.D.	−	−	−	−	−	7.25	6.25	2	−	≤1, S	≤0.25, S	N.D.	N.D.	N.D.

^a^ Determined by the eazyplex^®^ Superbug CRE. ^b^ AST performance and result interpretation according to the EUCAST guideline. ^c^ CTA: cefotaxime, MER: meropenem, CTV: ceftazidime/avibactam, AZA: aztreonam/avibactam, CID: cefiderocol. ^d^ N.D.: not determined.

## Data Availability

Data form sequencing analyses are openly available at the European Nucleotide Archive (ENA). Other datasets analysed in this study are available upon reasonable request from the corresponding author.
